# Novel Anthropometry-Based Calculation of the Body Heat Capacity in the Korean Population

**DOI:** 10.1371/journal.pone.0141498

**Published:** 2015-11-03

**Authors:** Duong Duc Pham, Jeong Hoon Lee, Young Boum Lee, Eun Seok Park, Ka Yul Kim, Ji Yeon Song, Ji Eun Kim, Chae Hun Leem

**Affiliations:** Department of Physiology, University of Ulsan College of Medicine, 88 OlympicRo 43-gil Songpa-gu, Seoul, Republic of Korea; University of Alabama at Birmingham, UNITED STATES

## Abstract

Heat capacity (HC) has an important role in the temperature regulation process, particularly in dealing with the heat load. The actual measurement of the body HC is complicated and is generally estimated by body-composition-specific data. This study compared the previously known HC estimating equations and sought how to define HC using simple anthropometric indices such as weight and body surface area (BSA) in the Korean population. Six hundred participants were randomly selected from a pool of 902 healthy volunteers aged 20 to 70 years for the training set. The remaining 302 participants were used for the test set. Body composition analysis using multi-frequency bioelectrical impedance analysis was used to access body components including body fat, water, protein, and mineral mass. Four different HCs were calculated and compared using a weight-based HC (HC_Eq1), two HCs estimated from fat and fat-free mass (HC_Eq2 and HC_Eq3), and an HC calculated from fat, protein, water, and mineral mass (HC_Eq4). HC_Eq1 generally produced a larger HC than the other HC equations and had a poorer correlation with the other HC equations. HC equations using body composition data were well-correlated to each other. If HC estimated with HC_Eq4 was regarded as a standard, interestingly, the BSA and weight independently contributed to the variation of HC. The model composed of weight, BSA, and gender was able to predict more than a 99% variation of HC_Eq4. Validation analysis on the test set showed a very high satisfactory level of the predictive model. In conclusion, our results suggest that gender, BSA, and weight are the independent factors for calculating HC. For the first time, a predictive equation based on anthropometry data was developed and this equation could be useful for estimating HC in the general Korean population without body-composition measurement.

## Introduction

Energy transfer, temperature regulation, and human response to cold and heat exposure have been intensively investigated. It had been questioned why the human body needs new food daily as a source of energy input and how the energy is manipulated. Energy input would be transformed into two components, i.e. the storage and the loss. The energy that is released from the body includes the heat loss (H_loss_) and the work performed by the body [1]. The body consumes energy for maintaining its homeostasis. In the resting state, more than 75% of the energy expenditure is transformed into heat and is the so-called heat production (H_prod_). Heat released by the metabolic process warms up the body tissue and increases the core and the skin temperature. At rest, almost all H_prod_ is released via dry H_loss_ in which radiative heat loss, convective and conductive heat loss, respiratory heat loss, and evaporative heat loss account for 54–60%, 25%, 14%, and 7% of total H_prod_, respectively [2]. During exercise, when the body temperature (BT) increases up to a particular threshold, it alerts the preoptic/anterior hypothalamus to initiate heat dissipation by stimulating cutaneous vasodilation and sweating. At a given H_prod_, individual responses of the skin blood flow and the evaporation differ at BT threshold [3]. In particular, the heat stress circumstance occurs when H_prod_ is not completely released, and the heat-storage content (heat load, H_load_):

$$H_{load}=H_{prod}-H_{loss}$$ (Eq1)

produces an increase in body core temperature. In terms of BT, heat capacity (HC) is an important factor that determines the magnitude of temperature change in the body at a given H_load_. The investigation of HC dates back to the eighteenth century with experiments on the HC of animals performed by Crawford (cited by Kakitsuba) [4]. The most commonly used value of the average specific HC (S_p_HC), the HC per mass, of the human body was proposed as 0.83 kcal·kg^-1^·°C^-1^by Sentor in the nineteenth century (cited by Kakitsuba) [4]. The value of 0.83 kcal·kg^-1^·°C^-1^ has been widely used until the present despite the criticisms that the value overestimates the actual HC in estimating the heat-storage content [2, 5]. It has been revealed that S_p_HC of body components are not identical and the HC equation using S_p_HC for particular body components was more precise [4–9]. Based on S_p_HC (1.88 kcal·kg^-1^·°C^-1^for fat and 3.72 kcal·kg^-1^·°C^-1^for fat-free mass), as cited by Minard [6], Webb developed an equation and reported a S_p_HC as 0.837 and 0.669 kcal·kg^-1^·°C^-1^ for those who have a fat proportion of 12 and 50% of fat, respectively [7]. Using the same approach, Blaxter provided slightly different values for the same calculation as 0.765 and 0.652 kcal·kg^-1^·°C^-1^ [8]. Using a two-compartment model (fat and fat-free mass), Havenith created an equation for HC in which the S_p_HC for fat tissue was 2.51 kj·kg^-1^·°C^-1^(0.60 kcal·kg^-1^·°C^-1^) and the S_p_HC for fat-free mass was 3.65 kj·kg^-1^·°C^-1^ (0.872 kcal·kg^-1^·°C^-1^) [10, 11], whereas a four-element model proposed S_p_HC for water, fat, protein, and mineral tissues as 1, 0.507, 0.299, and 0.201 kcal·kg^-1^·°C^-1^, respectively [2]. Although the HC equation based on body-composition-specific HC can predict the heat storage more precisely than an average S_p_HC for the whole body, body composition analysis is not available for general population. H_load_ and H_loss_ should be equal so as to maintain the constant BT. H_loss_ depends on the body surface area (BSA) and the temperature gradient from the body to the environment [12, 13]. H_load_ increases the BT depending on the HC of the body. Therefore, BSA and HC may be correlated to each other. Our study attempted to compare the previously known HC estimating equations in order to investigate the relationship between HC and the anthropometric variables, including weight and BSA, to derive a predictive equation for HC with anthropometry data for the first time.

## Materials and Methods

### Study Participants

The study participants were apparently healthy volunteers aged from 20 to 70 years and who attended the project intending to develop the Korean Constitution Multicenter Bank, that was described elsewhere [14, 15]. Six hundred participants were randomly selected by random sampling using the replacement method from the pool of 902 participants that has been collected at Asan Medical Center from 2010 to 2015 for the training set. The remaining 302 participants were used as the test set for validation analysis. The study and the written consent procedure were approved by the Institutional Review Board of the Asan Medical Center and all participants provided the written consents for this study.

### Measurements

Standing height and weight were assessed using a digital scale. BSA was calculated using the DuBois and DuBois EF formula [16]. Body components including body-fat mass, protein mass, mineral mass, and total body water were measured by multi-frequency bioelectrical impedance analysis (BIA) using the InBody 720 body composition analyzer (Inbody, Seoul, Korea).

HC was estimated using four equations including HC_Eq1 which was based on the widely used average S_p_HC (0.83 kcal·kg^-1^·°C^-1^) and body weight (BW) [2], HC_Eq2 which was based on Webb’s equation [7], HC_Eq3 which was based on Havenith’s equation [10, 11], and HC_Eq4 which was based on a four-component model [2].

$$\text{HC}\;(\text{kcal}\cdot {{}^{\circ}\text{C}}^{-1})=0.83\;\times \text{Weight}$$ (HC_Eq1)

$$\text{HC}\;(\text{kcal}\cdot {{}^{\circ}\text{C}}^{-1})=\left[\left(\text{Fat mass}\right)\times 0.449+\left(\text{Lean mass}\right)\times 0.889\right]$$ (HC_Eq2)

$$\text{HC}\;(\text{kcal}\cdot {{}^{\circ}\text{C}}^{-1})=\left[\left(\text{Fat mass}\right)\times 0.600+\left(\text{Lean mass}\right)\times 0.872\right]$$ (HC_Eq3)

$$\text{HC}\;(\text{kcal}\cdot {{}^{\circ}\text{C}}^{-1})=\left[\begin{array}{c} \left(\text{Fat mass}\right)\times 0.507+\left(\text{Protein}\right)\times 0.299+\\ \left(\text{Water}\right)\times 1+\left(\text{Mineral}\right)\times 0.201 \end{array}\right]$$ (HC_Eq4)

### Statistical analysis

Pearson correlation coefficient and difference were calculated to determine the agreement between HCs estimated from the four equations. HC_Eq4 which was based on a four-component model was selected to perform regression analysis with weight, BSA, and other confounding factors. A predictive model for HC-based anthropometry data was developed. The validation analysis for the predictive model and the HC estimated by the selected equation was performed using Bland-Altman analysis. Data were analyzed using the R program version 3.2.1. Statistical significant level was set as a p value <0.05.

## Results

**Fig 1:**
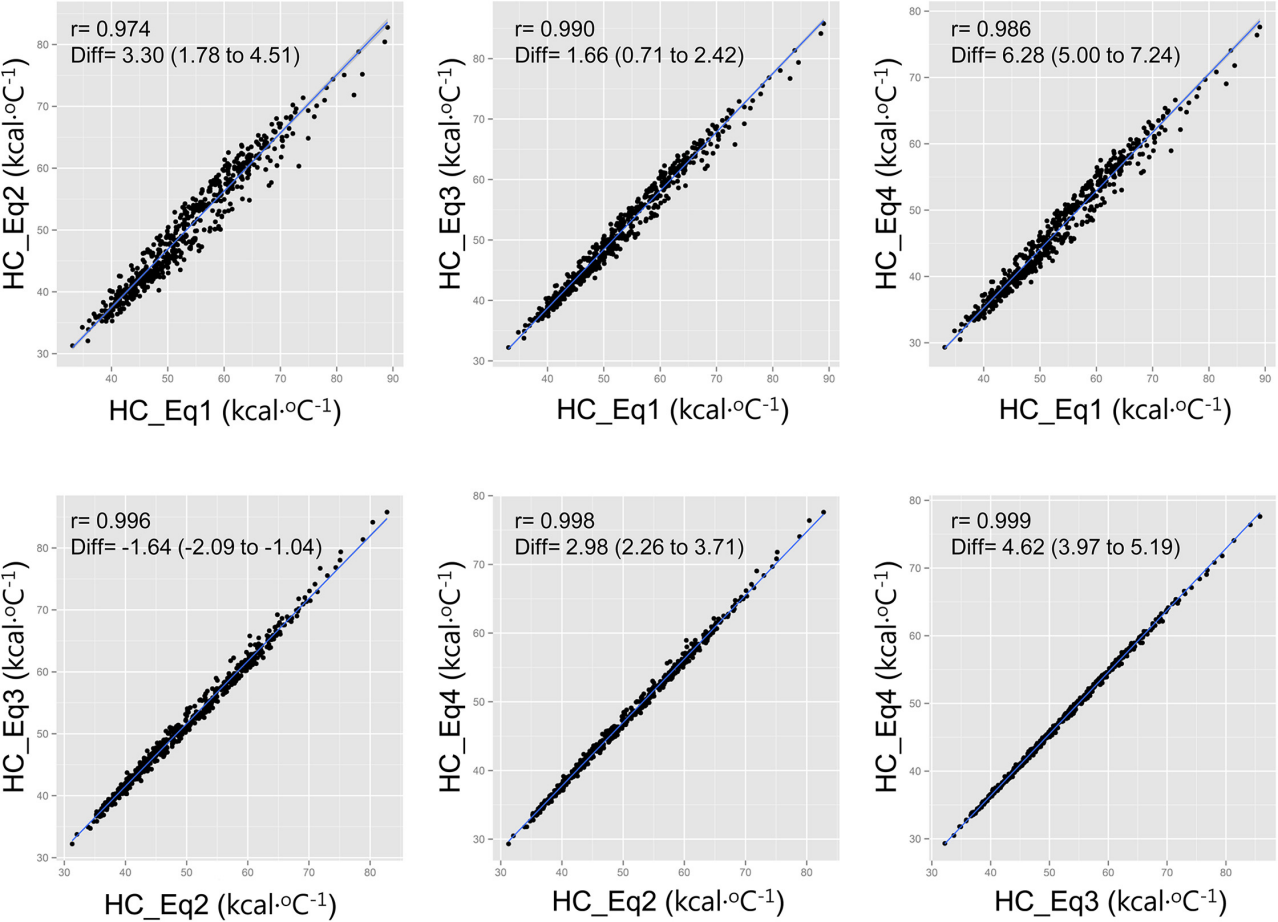
Correlation between the heat capacity (HC) values estimated by four equations. HC_Eq1, heat capacity calculated based on the widely used average specific heat capacity (0.83 kcal·kg^-1^·°C^-1^) and body weight; HC_Eq2, heat capacity calculated based on Minard’s equation; HC_Eq3, heat capacity calculated based on Havenith’s equation; and HC_Eq4, heat capacity calculated based on four components model. r, correlation coefficient; Diff, mean and 95% confident interval of difference between the two HC values (x—y).

The training set included 282 men and 318 women, whereas the test set was composed of 138 men and 164 women. There was no difference in the demographic data, body composition characteristics or HC among the training and test sets (Table 1). Fig 1 shows the correlations between each HC equation. In general, the HC equations were well-correlated to each other. Equations based on two components (HC_Eq2 and HC_Eq3) and four components (HC_Eq4) were strongly correlated (r = 0.996 to 0.999, mean difference = 1.64 to 4.62 kcal·°C^-1^), whereas equations using the average S_p_HC for the whole body overestimated HC compared to the other equations (r = 0.974 to 0.990, mean difference = 1.66 to 6.28 kcal·°C^-1^). The difference among HCs estimated by equations based on body composition data was lowest between HC_Eq4 and HC_Eq2. Because HC_Eq4 originated from more precise body composition analysis, further analysis regarding the relationship between HC and the anthropometric indices was performed using HC_Eq4.

**Table 1:** Demographic, body composition, and heat capacity by gender in the training and test sets.

	Training set (n = 600)		Test set (n = 302)	
	Men (n = 282)	Women (n = 318)	Men (n = 138)	Women (n = 164)
Age (yrs)	34.6 (10.9)	38.6 (11.5)	34.1 (10.6)	39.4 (12.2)
Weight (kg)	72.2 (10.1)	56.8 (7.6)	71.9 (10.0)	57.0 (7.4)
Height (cm)	173.0 (5.7)	159.7 (5.3)	173.2 (5.6)	159.8 (5.6)
BMI (kg·m^2^)	24.1 (2.9)	22.3 (3.0)	23.9 (3.0)	22.3 (2.9)
BSA (m^2^)	1.9 (0.1)	1.6 (0.1)	1.9 (0.1)	1.6 (0.1)
Total body water (kg)	41.8 (4.6)	29.2 (2.9)	41.7 (4.3)	29.1(3.0)
Body fat mass (kg)	15.3 (5.9)	17.0 (5.3)	15.1 (6.3)	17.3 (5.4)
Protein mass (kg)	11.3 (1.3)	7.8 (0.8)	11.3 (1.2)	7.8 (0.8)
Mineral mass (kg)	3.8 (0.5)	2.8 (0.3)	3.8 (0.4)	2.8 (0.3)
HC_Eq1 (kcal·°C^-1^)	59.8 (8.4)	47.1 (6.3)	59.6 (8.3)	47.3 (6.1)
HC_Eq2 (kcal·°C^-1^)	57.4 (7.1)	43.0 (4.9)	57.2 (6.8)	43.1 (4.8)
HC_Eq3 (kcal·°C^-1^)	58.8 (7.6)	44.9 (5.4)	58.6 (7.3)	45.0 (5.3)
HC_Eq4 (kcal·°C^-1^)	53.7 (6.8)	40.7 (4.8)	53.5 (6.5)	40.8 (4.7)

Data are presented as mean (SD). BMI, body mass index; BSA, body surface area; HC_Eq1, heat capacity calculated based on the widely used average specific heat capacity (0.83 kcal·kg^-1^·°C^-1^) and BW; HC_Eq2, heat capacity calculated based on Minard’s equation; HC_Eq3, heat capacity calculated based on Havenith’s equation; and HC_Eq4, heat capacity calculated based on a four-component model.

**Fig 2:**
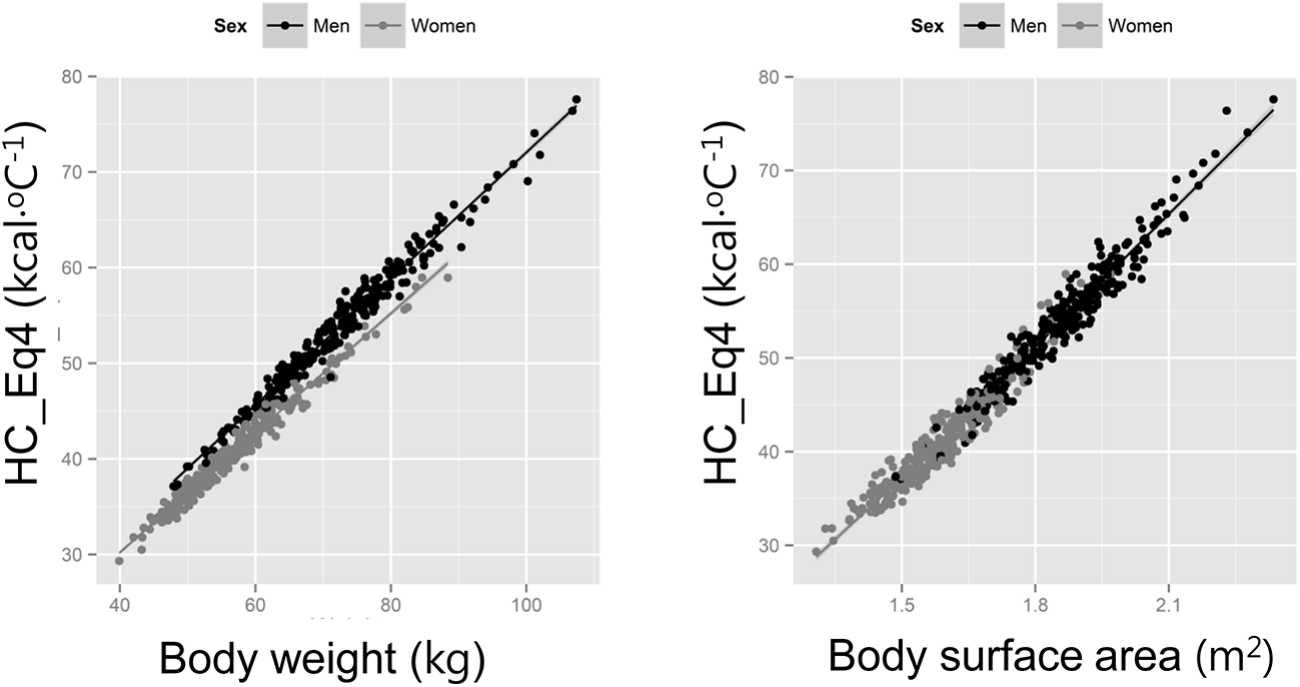
Relationship between heat capacity (HC) estimated using the equation based on the four-component model (HC_Eq4) and weight (a) and body surface area (b) for men (black dots) and for women (grey dots).

The relationship between BW versus HC_Eq4 and BSA versus HC_Eq4 could be described by a linear regression model (HC_Eq4 versus BW, r = 0.986, 0.988, and 0.986 for the total, men, and women, respectively; HC_Eq4 versus BSA, r = 0.985, 0.974, and 0.955 for the total, men, and women, respectively). In Fig 2, the correlation coefficients between HC_Eq4 and weight were high, however, there was a clear gender difference. The gender difference was attenuated between HC_Eq4 and BSA. This pattern was also found in the same analysis for HC_Eq2 and HC_Eq3 (S1 Fig). Table 2 shows the results of the multivariate regression analysis. In the case of model 1 adjusted according to age, gender, and BW, all of the factors were significantly associated. In the case of model 2 adjusted according to age, gender, and BSA, all of the factors were also significantly associated for predicting HC, however, the gender differences were clearly attenuated and the coefficient for female was decreased from -2.96 to -0.36. In the case of model 3 adjusted with age, gender, BW, and BSA, age was not associated with HC whereas gender, BW, and BSA, were significantly associated. Interestingly, although BSA and BW was strongly correlated (r = 0.97), they contributed independently in predicting HC. After adjusting for BW and BSA, women on the average had lower HC than men by 1.96 kcal·°C^-1^ (95%CI, 1.76 to 2.16 kcal·°C^-1^). Three factors, including gender, BW, and BSA, explained more than 99% of the variation of HC estimated by HC_Eq4 among individuals (Table 2). The predictive model for HC with anthropometry data of the 600 study patients was HC (kcal ⋅ °C^−1^) = 14.29 × BSA (m^2^) + 0.46 × BW (kg) − 1.96 (if women) − 6.06.

**Table 2:** Multivariate regression analysis for predicting HC_Eq4.

	Regression coefficient (95% CI)	Coefficient of determination (R^2^)
Model 1: Predictors are age, gender, and body weight		
Intercept	7.544 (6.878–8.209) ***	0.988
Age (yrs)	-0.014 (-0.021 to -0.008) ***	
Gender (Female)	-2.962 (-3.159 to -2.765) ***	
Body weight (kg)	0.646 (0.638–0.654) ***	
Model 2: Predictors are age, gender, and body surface area		
Intercept	-33.475 (-35.361 to -31.589) ***	0.972
Age (yrs)	0.031 (0.020 to 0.041) ***	
Gender (Female)	-0.359 (-0.711 to -0.008) *	
BSA (m2)	46.445 (45.478 to 47.413) ***	
Model 3: Predictors are age, gender, body weight, and body surface area		
Intercept	-6.048 (-7.718 to -4.379) ***	0.993
Age (yrs)	0.000 (-0.006 to 0.006)	
Gender (Female)	-1.961 (-2.160 to -1.762) ***	
Body weight (kg)	0.460 (0.438 to 0.483) ***	
BSA (m2)	14.299 (12.640 to 15.958) ***	

*p<0.05,

***p<0.001

**Fig 3:**
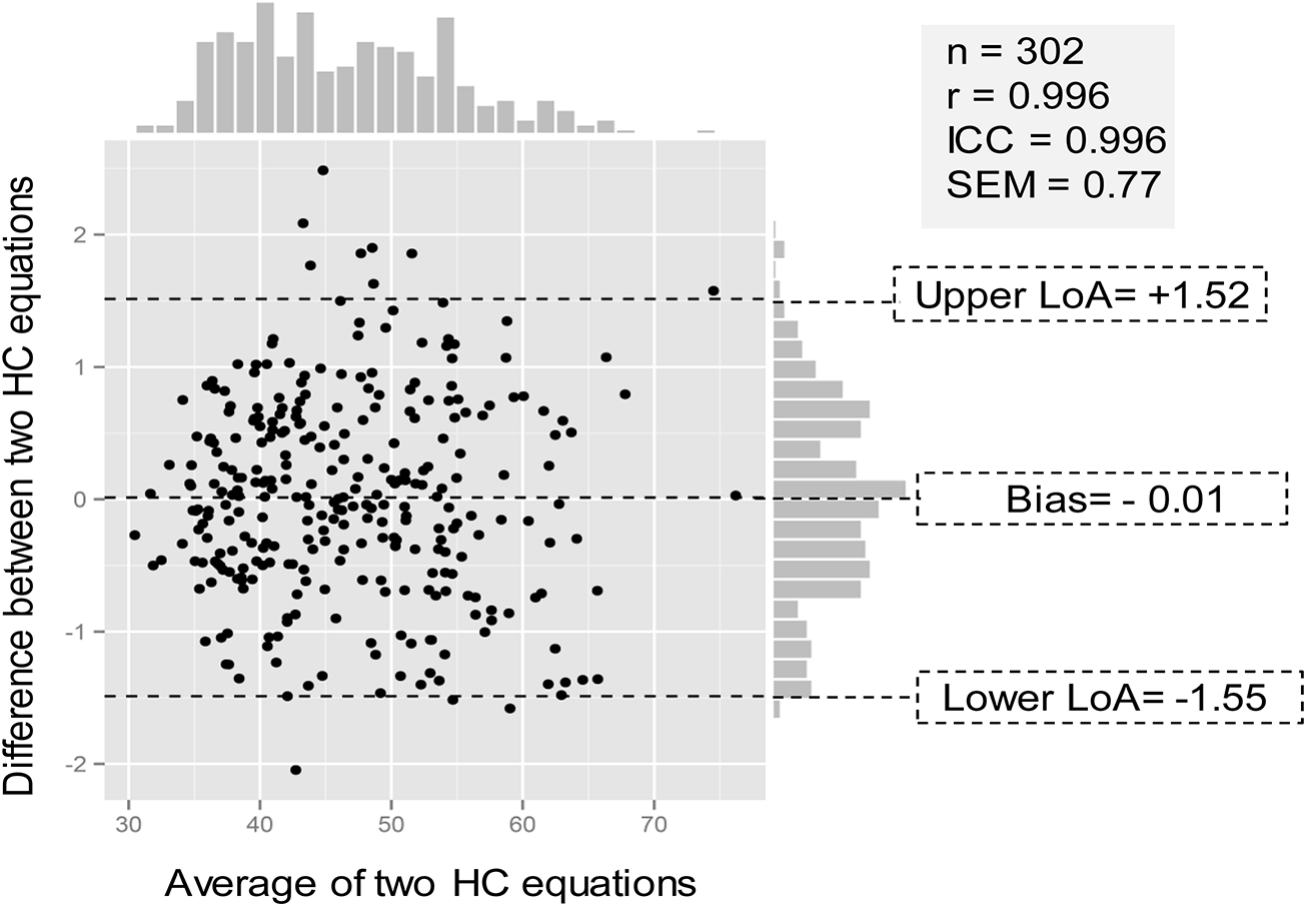
Bland Altman plot with marginal histograms for the agreement between the predictive model and HC_Eq4 in the test set. Bias, mean of differences between the two HC equations, the predictive model and HC_Eq4; Upper LoA, upper level of limits of agreement = mean of differences + 1.96 standard deviation; Lower LoA, lower level of limits of agreement = mean of differences—1.96 standard deviation; r, Pearson correlation coefficients; ICC, Intraclass correlation coefficient; SEM, standard error of measurement.

As for the analysis of concordance on the test set, the predictive model for HC with anthropometry data showed extremely highly satisfactory values (r = 0.996; ICC = 0.996). The predictive model also had a low standard error of measurement (SEM = 0.77 kcal·°C^-1^) with the limits of agreement varying approximately±1.5 kcal·°C^-1^ (Fig 3). The final predictive model for HC with anthropometry data from all of the study patients was HC (kcal ⋅ °C^−1^) = 14.482 × BSA (m^2^) + 0.456 × BW (kg) − 1.996(if women) − 6.064 (r = 0.996, S1 Table.)

## Discussion

Although HC plays an important role in the process of heat balancing, very few studies pay attention on investigating an accurate and simple tool for calculating HC. In previous studies, HC has been popularly calculated by multiplying BW to the average S_p_HC, 0.83 kcal·kg^-1^·°C^-1^. However, it has been revealed that the equation using the average S_p_HC overestimated the actual human body HC [4–9]. In concordance with that, in the present study the HC equation using the average S_p_HC provided a higher value compared with those using body composition data, particularly with that using the four components model. We also found that gender interacted more strongly in the relationship between HC and weight, but unsteadily in the relationship between HC and BSA. In addition, BSA contributed to HC as an independent factor in addition to weight. The model that includes both BW and BSA appears to be better than that containing each variable alone. The model composed of weight, BSA, and gender appeared to be extremely well fitted for predicting HC without any information regarding body composition. With the 99% variation of HC which could be explained by the model with gender, weight, and BSA, we are able to correctly estimate HC without using complicated body component measurement. The validation analysis showed that the predictive model was reliable and sufficient to be applied for estimating HC. Anthropometry-based calculation of HC is a very useful feature in many other fields, including sports science, architectural engineering, environment physiology, etc.

Our study is the first to date to attempt to investigate the relationship between BSA and HC. Heat load increases the BT depending on HC. Heat loss depends on the temperature gradient between the BT and the ambient temperature and BSA. Both heat load and heat loss should be the same for the maintenance of BT. Therefore, if the ratio of the change of BT and the temperature gradient is relatively constant, the HC and BSA should be linearly correlated with each other. Our results very strongly demonstrate this possibility. The relationship between weight and HC was highly correlated in our results, however, there were clear gender differences. Interestingly, the gender differences were greatly attenuated in the relationship between BSA and HC. These results suggest the possibility that the human body during its development adaptively matches HC and BSA to each other in order to regulate the BT.

The relationship of BW, BSA, and HC may shed some light on the heat flow in the body. During prolonged physical exercise, BW may be remarkably reduced due to dehydration while the BSA remains constant [17]. That consequently induces the reduction of HC, increases the temperature load, and enhances the heat-related damage. All of our study participants were healthy and without any known disease. The linear relationships between HC and BSA were observed in a healthy population in which all of the participants were considered as normal in their thermoregulation response and in their capacity to balance the heat flow. We assume that the balance between HC and BSA may be skewed in unhealthy status patients and which might be related to a thermoregulation disorder. This could be a potential study hypothesis in future studies.

Our findings should be interpreted in the context of their strength and limitations. The present study enrolled a relatively high number of participants with a wide age range. However, because validation analysis was performed on the estimated HC values, the predictive model should be confirmed with measured values. Furthermore, because there were fewer patients aged in their 60s and no patient was 70 or older, the findings should be cautiously applied in the elderly population. It has been widely accepted that multi-frequency BIA is valid tool to estimate body composition, particularly in predicting lean mass, fat mass, and total body water [18–22]. Evidence also revealed that multi-frequency BIA is a useful approach for body somatic protein store [23] and body bone mineral content estimation [24]. In addition, because proportion of protein and mineral mass is relatively low in contribution to BW, potential bias of these body components measurement in predicting body HC seems to be not crucial. Since the body fat content strongly affects the HC, the bias from our new equation may be caused by the fat content. We found the fat mass or the % fat mass correlated with the difference in predictive model HC and HC_Eq4, however, the estimation bias was relatively low and less than ± 1 kcal·°C^-1^ per 10 kg fat mass or per 10% fat mass (S2 Fig). Therefore the new equation can be applied within our data range of fat mass or % fat mass and should be employed with care for those who have extremely low or high fat mass.

In conclusion, our results suggest that gender, BSA, and weight are the independent factors for calculating HC. For the first time, a predictive equation based on anthropometry data was developed and this equation could be useful in estimating HC in the general population without body composition measurements.

## Supporting Information

S1 Fig Relationship between heat capacity (HC) estimated by the equation HC_Eq1, HC_Eq2, and HC_Eq3 versus weight and body surface area for men (black dots). (TIF)

S2 Fig Relationship between difference in predictive model HC and HC_Eq4 versus fat mass and proportion of body fat (using whole data, n = 902). (TIF)

S1 Table Multivariate regression analysis for predicting HC_Eq4 (Data n = 902). (DOCX)
