# Supplementary figures and images for: Novel Anthropometry-Based Calculation of the Body Heat Capacity in the Korean Population
Source: PLoS One. 2015 Nov 3;10(11):e0141498. doi: 10.1371/journal.pone.0141498 (PMC4631517; doi:10.1371/journal.pone.0141498)

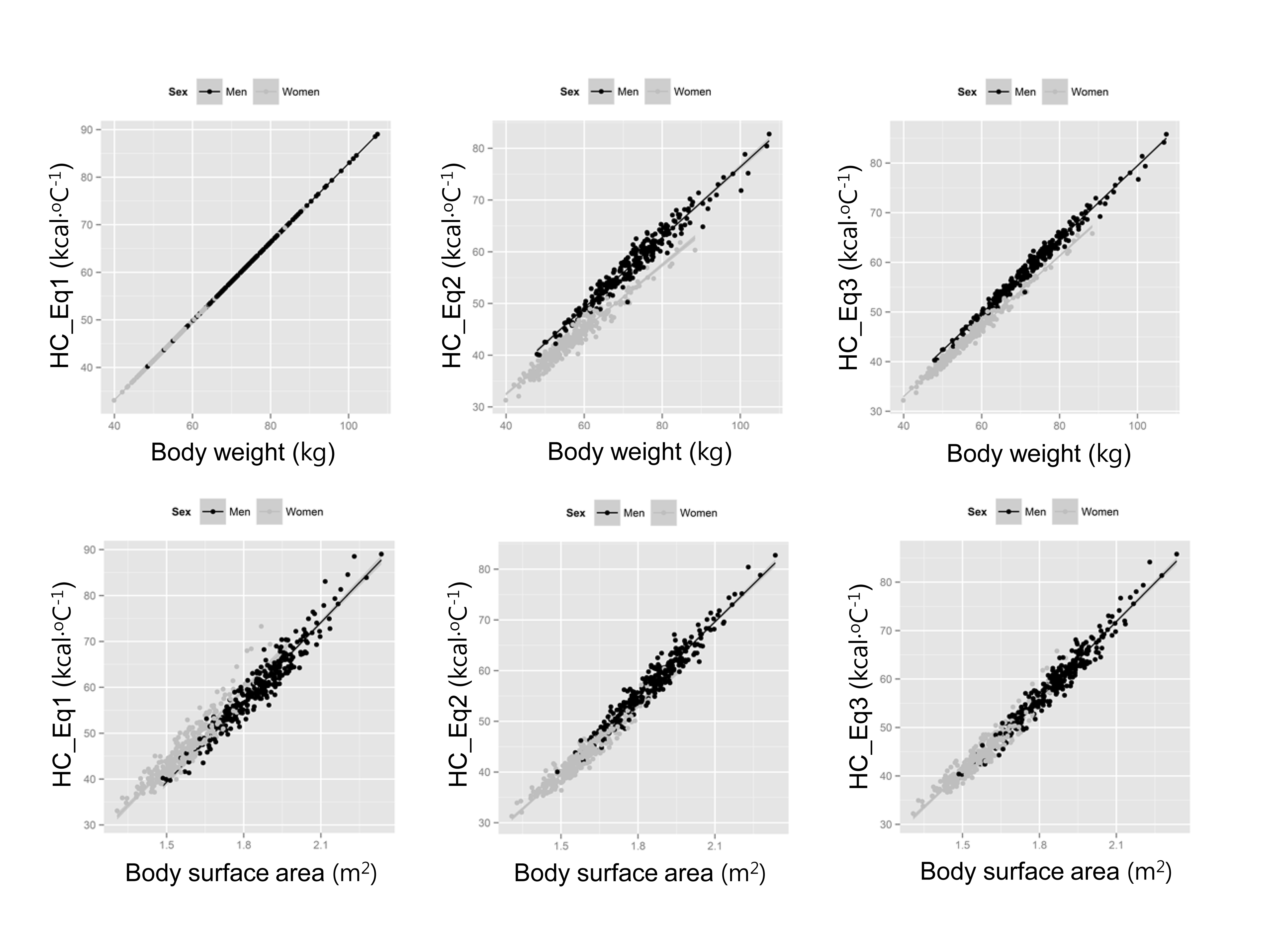

Supplement: S1 Fig — (TIF) [file pone.0141498.s001.tif]

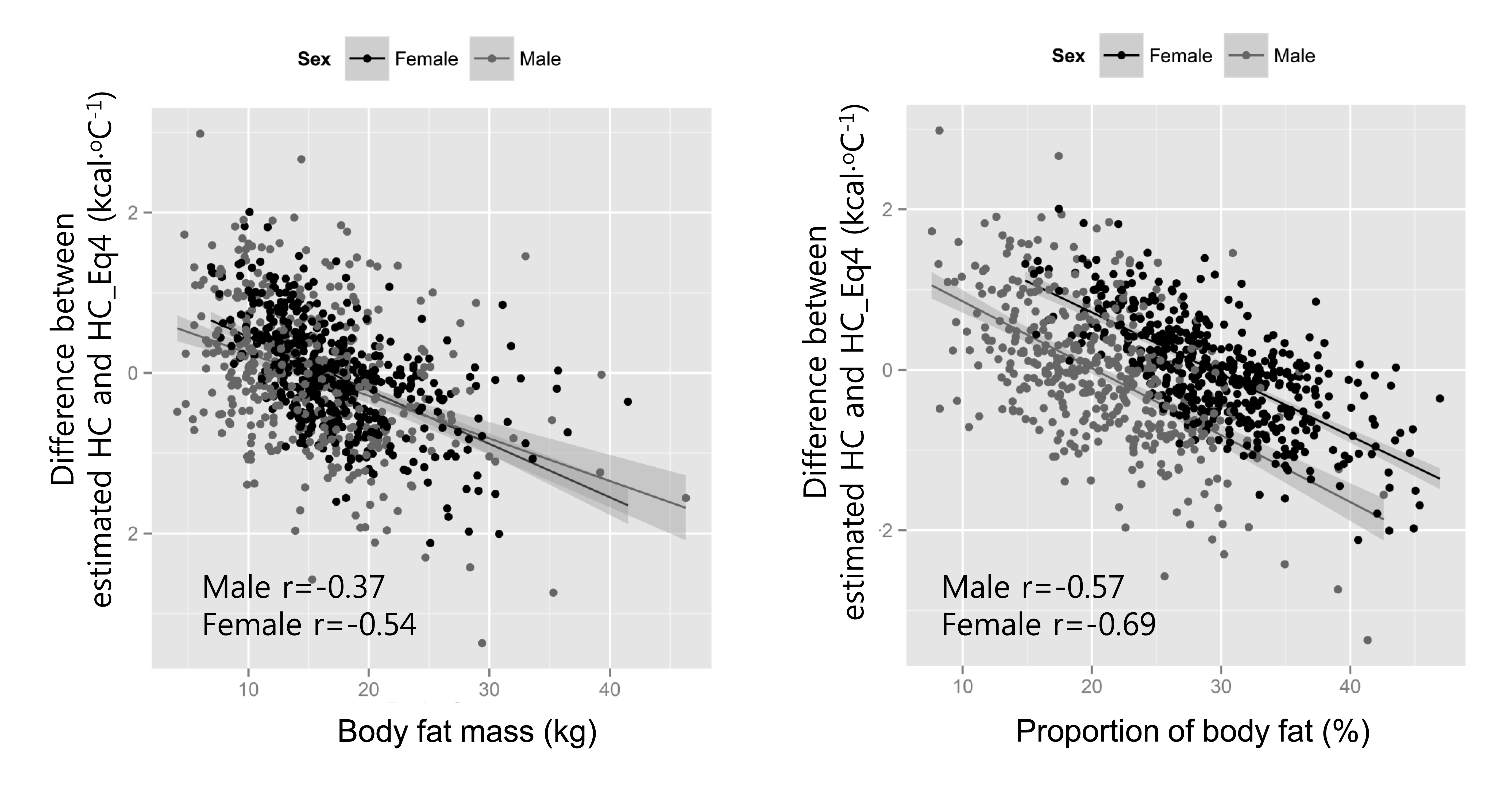

Supplement: S2 Fig — (TIF) [file pone.0141498.s002.tif]
