# Supplementary material for: Novel Anthropometry-Based Calculation of the Body Heat Capacity in the Korean Population
Source: PLoS One. 2015 Nov 3;10(11):e0141498. doi: 10.1371/journal.pone.0141498 (PMC4631517; doi:10.1371/journal.pone.0141498)
Supplement: S1 Table — (DOCX) [file pone.0141498.s003.docx]

**1S Table.** Multivariate regression analysis for predicting HC_Eq4 **(Data n=902)**

|  | **Regression coefficient (95% CI)** | **Coefficient of determination (R^2^)** |
| --- | --- | --- |
| **Model 1: Predictors are age, gender, and body weight** | | |
| **Intercept** | 7.882 (7.345 to 8.419) *** | 0.989 |
| **Age (yrs)** | -0.019 (-0.024 to -0.014) *** |  |
| **Gender (Female)** | -2.995 (-3.156 to -2.834) *** |  |
| **Body weight (kg)** | 0.643 (0.637 to 0.650) *** |  |
| **Model 2: Predictors are age, gender, and body surface area** | | |
| **Intercept** | -33.046 (-34.591to -31.501)*** | 0.971 |
| **Age (yrs)** | 0.030 (0.022 to 0.039)*** |  |
| **Gender (Female)** | -0.428 (-0.716 to -0.139)*** |  |
| **BSA (m2)** | 46.200 (45.402 to 46.998)*** |  |
| **Model 3: Predictors are age, gender, body weight, and body surface area** | | |
| **Intercept** | -5.614 (-6.970 to -4.259)*** | 0.992 |
| **Age (yrs)** | -0.004 (-0.008 to 0.001) |  |
| **Gender (Female)** | -2.006 (-2.169 to -1.843)*** |  |
| **Body weight (kg)** | 0.460 (0.442 to 0.478)*** |  |
| **BSA (m2)** | 14.138 (12.796 to 15.481)*** |  |
| **Model 4: Predictors are gender, body weight, and body surface area** | | |
| **Intercept** | -6.064 (-7.305 to -4.823) *** | 0.993 |
| **Gender (Female)** | -1.996 (-2.158 to -1.834) *** |  |
| **Body weight (kg)** | 0.456 (0.438 to 0.473) *** |  |
| **BSA (m2)** | 14.482 (13.206 to 15.759) *** |  |
